# Singing teaching as a therapy for chronic respiratory disease - a randomised controlled trial and qualitative evaluation

**DOI:** 10.1186/1471-2466-10-41

**Published:** 2010-08-03

**Authors:** Victoria M Lord, Phoene Cave, Victoria J Hume, Elizabeth J Flude, Amanda Evans, Julia L Kelly, Michael I Polkey, Nicholas S Hopkinson

**Affiliations:** 1 Royal Brompton & Harefield NHS Foundation Trust, Sydney Street, London SW3 6NP, UK; 2 National Heart and Lung Institute, Imperial College, Royal Brompton Campus, Fulham Rd, London SW3 6NP, UK

## Abstract

**Background:**

Despite optimal pharmacological therapy and pulmonary rehabilitation, patients with COPD continue to be breathless. There is a need to develop additional strategies to alleviate symptoms. Learning to sing requires control of breathing and posture and might have benefits that translate into daily life.

**Methods:**

To test this hypothesis we performed a randomised controlled trial, comparing a six week course of twice weekly singing classes to usual care, in 28 COPD patients. The experience of singing was assessed in a qualitative fashion, through interviews with a psychologist. In addition, we surveyed patients with chronic respiratory conditions who participated in a series of open singing workshops.

**Results:**

In the RCT, the physical component score of the SF36 improved in the singers (n = 15) compared to the controls (n = 13); +7.5(14.6) vs. -3.8(8.4) p = 0.02. Singers also had a significant fall in HAD anxiety score; -1.1(2.7) vs. +0.8(1.7) p = 0.03. Singing did not improve single breath counting, breath hold time or shuttle walk distance. In the qualitative element, 8 patients from the singing group were interviewed. Positive effects on physical sensation, general well-being, community/social support and achievement/efficacy emerged as common themes. 150 participants in open workshops completed a questionnaire. 96% rated the workshops as "very enjoyable" and 98% thought the workshop had taught them something about breathing in a different way. 81% of attendees felt a "marked physical difference" after the workshop.

**Conclusion:**

Singing classes can improve quality of life measures and anxiety and are viewed as a very positive experience by patients with respiratory disease; no adverse consequences of participation were observed.

**Trial Registration:**

Current Controlled Trials - ISRCTN17544114.

## Background

Chronic obstructive pulmonary disease (COPD) causes breathlessness, which can occur on exertion or, in some individuals, at rest. Although therapy including bronchodilators, pulmonary rehabilitation and oxygen can improve symptoms, the underlying pathology is largely irreversible, meaning that many patients remain limited despite optimal therapy [1,2].

The pattern of breathing typically adopted in COPD is caused by the presence of expiratory flow limitation which leads patients to adopt higher operating lung volumes - dynamic hyperinflation [3]. Although this pattern allows increased expiratory flow rates, this comes at the expense of a greater inspiratory load and mechanical disadvantage for the respiratory muscles. A rapid breathing pattern reduces expiratory time and will exacerbate this. The disparity between respiratory work and ventilatory output that develops is an important determinant of symptoms [4]. A range of non-pharmacological measures for breathlessness have been trialled in respiratory patients [5], including pursed lip breathing [6,7], yoga [8,9], singing [10] and laughter [11], all based on the premise that changing patients' pattern of breathing will improve symptoms

A limitation of some physiotherapy approaches is that by focusing attention on breathing pattern they may accentuate awareness of respiratory limitation [12]. Singing requires the development of skills in controlling posture and breath that might be transferrable to everyday life. Breathlessness is a complex sensation [13]; singing lessons might offer techniques that address both the sensory component - largely control of respiratory pattern to reduce hyperinflation, and the affective component - a conscious experience of using the breath for 'something positive'. In addition, there is some evidence that singing may have beneficial effects on wellbeing in healthy [14] and chronic disease populations [15,16].

To evaluate this further we used three approaches; firstly a randomized controlled trial of a six week course of singing training in COPD, secondly an evaluation of a program of open 'drop-in' singing lessons for patients with respiratory disease and thirdly a qualitative evaluation of patients' experience of participating in singing.

## Methods

### Randomized controlled trial

Patients with COPD, diagnosed according to the GOLD guidelines, who were attending respiratory clinics at the Royal Brompton Hospital, were invited to participate. The study was approved by the Royal Brompton, Harefield and National Heart and Lung Institute Research Ethics Committee and all subjects gave written informed consent. The trial was registered at Current Controlled Trials - ISRCTN17544114.

Demographics, height, weight, clinical history and spirometry (Microlab, CareFusion, Kent, UK,) were recorded. Participants completed the Hospital Anxiety and Depression (HAD) Questionnaire [17], St George's Respiratory Questionnaire [18], and the Short Form 36 Questionnaire [19]. Functional exercise capacity was assessed using the incremental shuttle walk test (ISWT). Time to recovery of oxygen saturation, Borg dyspnoea score and heart rate following the walk was documented [20].

Control of breathing was assessed using two measures. 1) Breath hold test - subjects held their breath from maximum inspiration [21,22]. 2) Single breath counting - subjects were instructed to breathe in and then count out loud in time with a metronome running at 60 beats/min[23]. Three attempts at each manoeuvre were made and the mean values recorded. Both techniques were in routine use in the physiotherapy department for the assessment of hyperventilation.

All subjects received a thirty minute standard session on breathing control and techniques to manage breathlessness, delivered by one of two senior respiratory physiotherapists involved in the study. Pursed lip breathing and nose breathing were also discussed in relation to managing episodes of shortness of breath. Each subject received a standard Royal Brompton Hospital "Help Yourself - physiotherapy for people with respiratory symptoms" booklet (Additional file 1) and was advised to practice the techniques at home.

At the end of the baseline session, patients were randomised to either the singing or control groups, using block randomization through consecutive sealed envelopes. The subjects in the singing group attended a hospital-based workshop, led by a singing teacher (PC), twice weekly for six weeks. Each session lasted approximately one hour and included teaching of posture, relaxation and vocal exercises. Tasks of increasing difficulty were given as the sessions progressed - described in more detail in additional file 2. Each subject was given homework and an accompanying CD of songs to practice at home. The teacher was unaware of the tests being used to assess breathing control, so that she would not 'teach to the test'. The control group had no further intervention.

At seven weeks follow-up, study participants were again assessed by the same respiratory physiotherapists, who were blinded to treatment allocation. All subjects were instructed not to tell the physiotherapist which group they had been allocated to. Control of breathing, quality of life and functional exercise capacity were measured as at baseline.

Statistical analysis was performed using StatView 4.0, using t-tests to compare change in outcome measures between patients and controls. A p value of < 0.5 was taken as significant.

### Evaluation of open singing workshops for respiratory patients

Alongside the randomized control study, we ran open, twice-weekly, 'Singing for Breathing™' workshops for patients with any respiratory condition who were at Royal Brompton Hospital either as inpatients, day cases or outpatients. Conditions included interstitial lung disease, cystic fibrosis, asthma, bronchiectasis and COPD. The sessions were advertised through posters in the hospital and by word of mouth to patients from the arts team, respiratory nurses, physiotherapists and other healthcare professionals.

Patients involved in the randomized control study were excluded from these sessions as were those where infection control issues existed. The open sessions were led by the same singing teacher as the randomised study and encompassed relaxation and posture education, as well as phonation exercises.

Patients attending these workshops for the first time were asked to complete an anonymous satisfaction questionnaire at the end (Additional file 3).

### Qualitative survey of patient experience of singing

Following completion of the randomized control study, a chartered counselling psychologist (AE), who had not otherwise participated in the study, interviewed a random sample of eight individuals who had been in the singing arm of the trial. The thirty minute interviews were structured and each subject was asked about 1) physical changes they had noticed. 2) Emotional changes they had noticed. 3) Behavioural changes they had noticed. 4) Any detrimental effects or negative experiences. Participants were assured that they could make negative comments if they wished and that their responses would be anonymous. The interview sheet is shown in additional file 4.

## Results

### Randomized controlled trial

**Figure 1:**
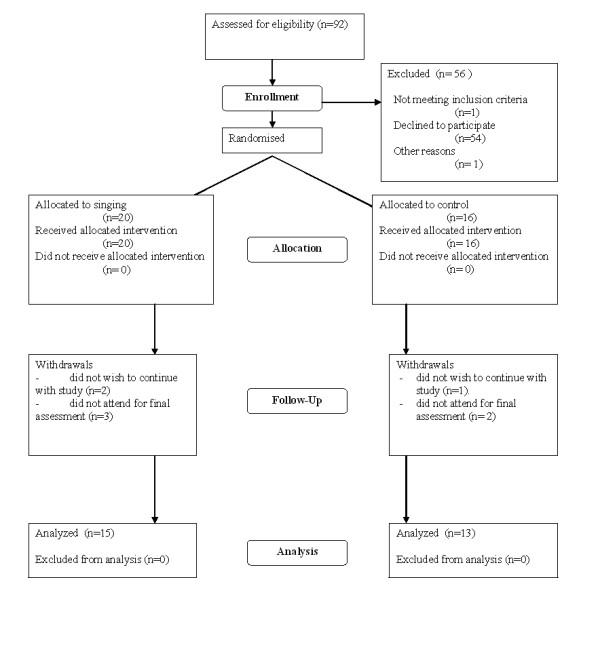
**CONSORT Flow Diagram**.

Participation in the study is described in the CONSORT diagram (Figure 1). Ninety two patients were approached to participate in the study. Fifty four did not wish to take part, one person who was already enrolled in another trial was excluded and one lived too far away to participate. Of the remaining thirty six patients, twenty were allocated to the singing group and sixteen to the control group. Of the singing group, two subjects withdrew from the study once the workshops had begun and three did not attend the final assessment once completing the workshops. In the control group, one withdrew and two did not attend the final assessment. Data are therefore presented for the 15 singers and 13 controls who completed the study. Of these, 6 attended 12 sessions, 4 attended 11, 2 came to 10 and 3 to 9.

The baseline characteristics of the singing and control groups were well matched (Table 1). Four of each group were on long term oxygen therapy. In singers, there was a significant improvement in HAD anxiety score -1.1(2.7) vs. +0.8(1.7) p = 0.03 and SF-36 physical component score +7.5(14.6) vs. -3.8(8.4) p = 0.02. Breath hold time actually increased in the control group relative to the singing group -0.3(6.9) sec vs. +5.3(5.7) sec (p = 0.03). There was no significant difference in single breath counting, functional exercise capacity or recovery times following ISWT (Table 2).

On direct questioning by the physiotherapist at the end of the study all participants in the singing group reported that they had found the workshops helpful, had practiced their singing at home and that they had found the singing helped them to control their breathing.

**Table 1:** Baseline characteristics

	Total n = 28	Singing n = 15	Control n = 13	P value
Age (yrs)	67.3 (8.1)	66.6 (9.3)	68.1 (6.8)	0.6
FEV_1 _% predicted	37.2 (18.6)	36.8 (15.4)	37.7 (22.4)	0.9
Breath hold time (s)	26.3 (11.1)	27.4 (11.7)	24.9 (10.5)	0.9
Single breath counting (n)	23.4 (6.9)	24.8 (7.7)	21.8 (5.7)	0.3
HAD - anxiety score	5.8 (2.9)	6.3 (3.1)	5.3 (2.6)	0.4
HAD -depression score	5.8 (3.1)	5.7 (2.8)	5.8 (3.6)	1.0
SGRQ Total	48.4 (14.5)	51.1 (14.8)	45.3 (14.0)	0.3
SF36 PCS	36.8 (18.4)	32.0 (14.0)	42.2 (21.8)	0.1
SF36 MCS	52.0 (17.9)	47.4 (14.8)	57.2 (20.1)	0.2
ISWT (m)	198.6 (131.3)	174.0 (137.2)	226.9 (123.3)	0.3
Subjective recovery (s)	124.2 (77.6)	115.9 (50.4)	133.8 (102.0)	0.6
O_2 _sat^n ^recovery (s)	77.7 (71.6)	73.5 (44.6)	82.6 (95.7)	0.7
HR recovery (s)	131.0 (89.5)	97.6 (40.8)	169.6 (114.3)	0.03*

FEV_1 _- forced expiratory volume in one second, HAD - hospital anxiety and depression score, SGRQ - St George's Respiratory Questionnaire, SF36 short form 36, PCS - physical component score, MCS - mental component score, ISWT - incremental shuttle walk test distance, HR heart rate. *p < 0.05 unpaired t test singing group vs control group.

**Table 2:** Changes from baseline

	Singing group n = 15	Control group n = 13	
Δ breath hold time (s)	- 0.3 (6.9)	+ 5.3 (5.7)	0.029*
Δ single breath counting	+ 0.3 (7.7)	+ 2.0 (2.7)	0.46
Δ HAD anxiety	- 1.1 (2.7)	+ 0.8 (1.7)	0.033*
Δ HAD depression	- 1.1 (2.5)	- 0.1 (1.7)	0.21
Δ SGRQ Total	- 1.1 (10.6)	- 0.4 (5.6)	0.81
Δ ISWT (m)	+ 26 (52.6)	+ 11.3 (83.0)	0.58
Δ ISWT subjective recovery (s)	+ 9.9 (60.7)	- 7.4 (81.7)	0.53
Δ O_2 _Sat^n ^recovery (s)	+ 47.3 (67.6)	+ 32.2 (124.7)	0.69
Δ HR recovery time (s)	+ 29 (63.8)	+ 19.4 (110.0)	0.78
Δ SF36 PCS	+ 7.5 (14.6)	- 3.8 (8.4)	0.02*
Δ SF36 MCS	+ 2.5 (20.9)	- 3.2 (10.5)	0.38

HAD - hospital anxiety and depression score, SGRQ - St George's Respiratory Questionnaire, ISWT - incremental shuttle walk test distance, HR heart rate, SF36 short form 36, PCS - physical component score, MCS - mental component score. *p < 0.05 unpaired t test singing group vs control group.

### Evaluation of open singing workshops for respiratory patients

150 patients attended the open workshops. 61% had never attended singing lessons or workshops previously. 96% rated the workshops as "very enjoyable" and 98% thought the workshop had taught them something about breathing in a different way. 81% of attendees felt a marked physical difference after the workshop.

92% of the participants said that they would like to attend a further singing workshop at the hospital. The remaining patients stated that the distance to the hospital would be the main reason for not attending.

### Qualitative survey of patient experience of the singing classes

Eight patients who had participated in the singing groups were interviewed. The effect of the group was described by all in mainly positive terms and no participants reported any negative effects. Positive effects occurred in two main areas: physical and general well-being.

Positive physical effects reported by the participants often related to the breathing training which was an integral part of the program, particularly "diaphragmatic breathing". This training brought a feeling of awareness and control which many of the participants found helped their breathing and eased symptoms of breathlessness. Singing training itself brought breathing control too; *"It has made my life easier; I would have liked this when I was first diagnosed", "I increased my out breath from 4 to 14 counts", "I started breathing much better, from the stomach", "The exercises, thinking about breathing and relaxing when I have (breathing) problems....this has been very useful", "I always felt better afterwards physically"*. Other positive physical effects described were an impact on lifestyle and functional ability; *"I have better posture now"; "Walking better, I go out more when it's not cold", "Now things are less of a chore, housework is no longer a struggle"*.

Beneficial effects relating to general wellbeing also emerged. Relating to mood/pleasure participants reported that; *"It was very enjoyable", "It opened up a new lease of life", "Emotionally......during singing it lifts you. I feel on top on the world. I also feel like that the day after. It makes COPD a lot easier to live with", "Its uplifting to sing...this diagnosis is gloomy so the psychological effect of the group is good"*.

A feeling of community/social support was also reported; *"Nice to have human contact, we achieved something together in the group", "I enjoyed the social contact it was great fun", "In six weeks we gelled as a group", "It felt good to be part of a team"*.

Achievement/efficacy - many participants had carried aspects of the courses into daily life. Many of them continued to sing songs and perform the exercises from the group using printed sheets or a CD. It seems that learning to sing in a group brought a sense of achievement. *"I do the exercises from the CD 2-3 times a week. I am looking for a local choir"; "I will keep it up; it's more enjoyable than other kinds of exercise"; "I learnt a lot, you are never too old to learn"; "The group taught me how and when to breathe; I got to relive my past (of singing) and get praise"*.

Others reported that participating in the group had motivated them to reengage with pulmonary rehabilitation classes, or to take up exercises such as swimming and walking. One was having private singing lessons and several expressed a desire for such groups to be ongoing and made more available to patients with respiratory problems.

## Discussion

The main findings of this study are that (1) the program of singing classes improved quality of life and anxiety but did not improve the control of breathing measures, or functional exercise capacity. (2) In interviews, patients who had participated in the trial reported benefits in their physical performance and general well-being as well as a sense of achievement and self-efficacy. (3) Individuals who chose to participate in the open singing sessions were overwhelmingly positive about the experience.

### Significance of findings

The idea that singing was beneficial for health goes back at least to the 19^th ^century [24]. Two previous studies have looked specifically at singing in COPD patients. Bonilha *et al *randomised patients to either singing lessons, or a handicraft class once a week for 6 months [10]. Although singing practice produced an acute increase in inspiratory capacity, implying a reduction in gas trapping, it did not alter basal dyspnoea index (BDI). SGRQ improved equally in both groups and the singing group had an improvement in maximum expiratory pressure. Exercise capacity was not assessed. Engen studied 7 patients with emphysema who attended twice weekly vocal instruction classes for 6 weeks [23]. There was no change in spirometry, inspiratory muscle strength or exercise capacity, but patients had an improvement in single breath counting, which was associated with a change from a 'clavicular' to a 'diaphragmatic' pattern of breathing. In that study there was no control group and the teacher was not blind to the outcome measures.

In the present study, anxiety score and physical component score of the SF36 improved and participants who were interviewed reported that they felt that the singing had been helpful in their everyday lives. HAD scores were not particularly high at baseline and patients were selected on the basis that they had symptomatic COPD and were able to attend, rather than because they were felt specifically to have a dysfunctional breathing pattern or psychological difficulties. It is possible that greater improvements might be found in particularly anxious sub-populations. The benefit in the physical rather than the mental component is consistent with the open workshop participants' reports that they felt physically different after the sessions.

The immediate benefits reported by patients participating in the open workshops and from the interviews with singing patients are consistent with the previous observation that singing has an acute effect of reducing gas trapping [10]. Most reported sustained physical benefits, specifically identifying the value of learning breathing control. Several participants also mentioned that they felt that it was important to keep up the vocal exercises and singing in order to maintain any gains which may suggest that a longer period is necessary.

The present study is also consistent with previous work that has found that singing training does not change, at least over the time courses explored to date, parameters such as exercise capacity [10,23]. We had expected that even if the singing class did not improve exercise tolerance it might hasten recovery, with patients adopting a more efficient breathing strategy. However, recovery time for heart rate, oxygen saturation or symptoms did not improve. Given the subjective benefits in physical sensation described by patients, the lack of change in exercise capacity is interesting. One possibility is that the improvements are entirely mediated in a psychological fashion. Since anxiety and depression are common and important co-morbidities in COPD [25,26] a novel approach to dealing with them could in any case be useful, particularly a non-drug treatment. An analogy would be the use of exercise prescriptions to treat depression [27]. Given the clear effects on wellbeing mentioned by the participants, one could also speculate that there might be benefits to efficacy and control in the absence of great physiological improvement. A patient's experience of their illness will be affected by a range of psychosocial factors in addition to their physical condition. All patients interviewed reported doing more singing and other pleasurable activities in their lives, suggesting that there may be long term benefits to participation in such a group. Consistent with this, singing has been used with some success in small studies to treat chronic pain where it seemed to improve coping [15] and after knee surgery [28].

A second possibility is that the 'dose' of singing was insufficient to alter conventional measures of breathing control or exercise capacity and that a longer or more intense period would be required to produce changes apparent in daily life. The singing teacher reported that she felt that after 6 weeks she was "only just starting to make progress" with many of the participants.

It is interesting that in the current study, patients reported that what was identified to them as a 'diaphragmatic' breathing pattern was helpful. This involves facilitating outward movement of the abdomen while reducing upper ribcage movement during inspiration. It has been thought to be helpful in some patients, but although a reduction in oxygen cost of breathing has been noted [29], the approach has been associated with both an increased sensation of breathlessness and reduced mechanical efficiency in severe COPD [30,31] and is not recommended for routine use in the recent British Thoracic Society/Association for Chartered Physiotherapist in Respiratory Care(BTS/ACPRC) guidelines on "Physiotherapy management of the adult, medical, spontaneously breathing patient" [12].

The improvement in breath hold time observed in the control group was unexpected. A strength of the study was that the singing teacher was blind to the outcome measures used so could not "teach to the test". The teacher speculated that the singing group may have learnt to take a more controlled or comfortable breath and therefore smaller breath in, leading paradoxically to a reduction in breath hold time. Hyperventilation has been shown to increase breath hold time in patients with respiratory disease which might also be relevant [22].

If the benefits of participating in a singing group are largely psychological, it would suggest that attention would need to be focused on the aspects of the singing that addressed this, or even on the choice of material, rather than focussing on particular nuances of technique. Singing is, of course, likely to be a therapy that suits some people and not others, and the benefits that accrue are likely to be greatest in those who enjoy the experience. This is to some extent different from pulmonary rehabilitation where the health benefits of exercise are generally accepted. As with pulmonary rehabilitation the physiological and psychological effects could be complementary.

A limitation of our study was the absence of an active control group. The previous paper of Bonilha et al. showed similar improvements of quality of life in patients submitted to singing training or handcraft artwork [10], so the observed improvements in anxiety and quality of life in the trial may have been due to regular contact with a social group rather than the singing specifically. However, in our study patients reported immediate positive effects on well being (probably because of a reduction in dynamic hyperinflation [10]) and this may therefore have reinforced participation in singing groups (as opposed to other 'social activities' which, although social have no physical effect), making the provision of singing classes a good strategy for reducing social isolation, a significant problem in chronic respiratory disease [32].

## Conclusions

Singing lessons improved anxiety and the physical component score of the SF36 but did not improve measures of breathing control, functional exercise capacity or recovery time. Participants reported that they found the singing beneficial and reported positive changes in their physical ability and wellbeing. It is likely that the effects of singing training will vary between individuals, but that it will be a positive experience for those who choose to take part. Further work is needed to quantify the magnitude and duration of improvement benefits, to set them in context against the resource implications of making singing groups for patients more widely available as a palliative therapy.

## Competing interests

The authors declare that they have no competing interests.

## Authors' contributions

NSH, VML, VJH, PC and MIP conceived the study; VML, PC, EJF, AE and JLK performed the study measurements. VML, VJH, AE and NSH performed the data analysis. VML wrote the first draft of the paper to which all authors subsequently made contributions. All authors read and approved the final manuscript.

## Pre-publication history

The pre-publication history for this paper can be accessed here:

http://www.biomedcentral.com/1471-2466/10/41/prepub

## Supplementary Material

Additional file 1 **Help Yourself - physiotherapy for people with respiratory symptoms"**. Standard booklet given to all patients participating in the trial. Click here for file

Additional file 2 **Singing for breathing - description of program**. Detailed description of singing program, selection of materials and conduct of classes. Click here for file

Additional file 3 **Open sessions satisfaction survey**. Survey used for participants to assess the open singing sessions. Click here for file

Additional file 4 **Structured interview template**. Template used to guide assessments by psychologists following the singing trial. Click here for file
