# Supplementary material for: Singing teaching as a therapy for chronic respiratory disease - a randomised controlled trial and qualitative evaluation
Source: BMC Pulm Med. 2010 Aug 3;10:41. doi: 10.1186/1471-2466-10-41 (PMC2920262; doi:10.1186/1471-2466-10-41)

Royal Brompton & Harefield NHS Trust

Help Yourself -
Physiotherapy for people with respiratory symptoms

Royal Brompton & Harefield NHS Trust

Royal Brompton Hospital
Sydney Street
London
SW3 6NP
tel: 020 7352 8121

Harefield Hospital
Hill End Road
Harefield Middlesex
UB9 6JH
tel: 01895 823 737

minicom: 01895 828534
website: www.rbh.nthames.nhs.uk

December 2004

Name: ______________________________

Physiotherapist: ______________________________

Contents

Sputum production	2
Chest clearance	2
Postural drainage	6
Breathlessness	9
Exercise and activity	13
Further information	15

Introduction

This booklet has been designed as a ‘how to help yourself’ guide, a source of information from which you will benefit if your lung condition gives you symptoms such as:

- breathlessness
- sputum production or retention
- reduced ability to exercise (exercise tolerance).

The information provided in this booklet should help you to remember what your physiotherapist has taught you.

Sputum Production

Normally, sputum is not a problem as it is produced by the lungs as a defence mechanism. Inhaled particles are transported out of the airways to keep them clean.

Sputum can become a problem if your condition involves the production of too much or if your disease affects the ability of your lungs to expel the mucus from your chest. This may involve part of or the whole of the lungs.

Sputum may become more of a problem when a range of possible invading bacteria, viruses or fungi cause a 'chest infection'. At this time you may notice the following changes:

- increasing amount of sputum
- change in sputum colour
- increasing frequency of cough
- possible increase in breathlessness.

It is advisable to seek advice from your GP when you have a chest infection. Medication may be provided to help your body defend itself against the infection.

You can also help yourself by using chest clearance techniques to expel the sputum from your chest.

Chest clearance

Why should I use chest clearance techniques?

Chest clearance will benefit your lungs by:

- expelling infected secretions and therefore helping to minimise futher infection
- reducing damage to the lung tissue caused by retained sputum
- helping to maintain lung volume and function and reduce breathlessness.

When should I use chest clearance techniques?

- Chest clearance should be used during chest infections to expel the excess secretions.
- It should also be used as a preventive treatment so that the risk of infection and lung damage is minimised.
- Each individual and each condition differs completely in the volume of sputum produced each day. It may vary from as little as a teaspoonful to maybe more than several teacups full. It is for this reason that you will need to ask your physiotherapist how often and for how long you should carry out these sessions.

What else can I do to make clearing my chest easier?

- Make sure that you are well hydrated to make sputum less sticky. Two litres of water each day is recommended, although it is advisable to reduce your caffeine intake by avoiding tea and coffee.
- If you have been prescribed 'bronchodilators', (medication which helps open up the airways), you may find it useful to take them about 15 minutes before clearing your chest to maximise the effect of the treatment.

Active Cycle of Breathing Technique (ACBT)

One technique that physiotherapists teach patients is the Active Cycle of Breathing Technique (ACBT). The diagram opposite will help you to understand this technique. Following the diagram you will find some short summaries which explain how to put the ACBT technique into practice.

Notes

Further information

Smoking

Some chest conditions are related to smoking and may make your breathlessness worse. If you wish to try to help your lungs by giving up smoking, your doctor or physiotherapist may be able to advise on the different options available. Some improvement to your lungs can be made by stopping smoking.

A telephone helpline is also available:

Quitline: 0800 002200   9.00am - 9.00pm

Further help

Further advice about your condition or on the information given in this booklet can be obtained from your physiotherapist. You can contact Harefield Hospital on tel: 01895 823737.

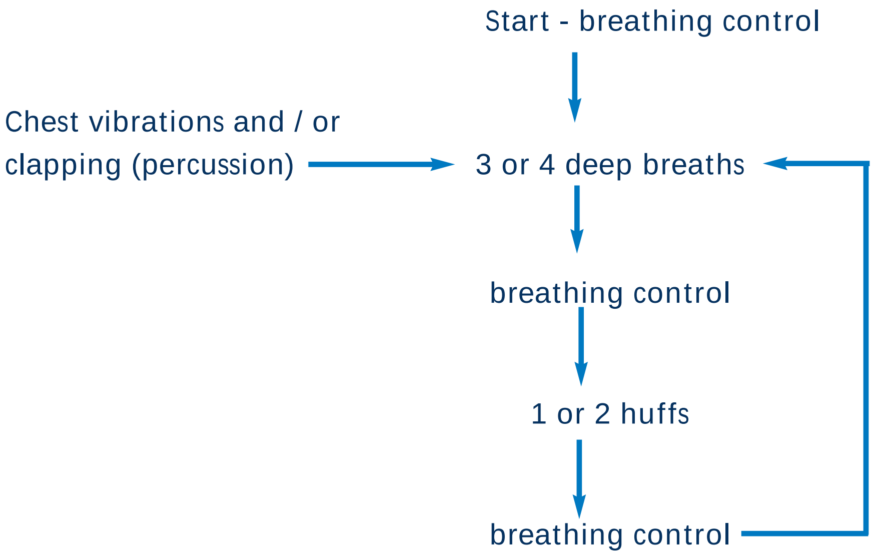

Breathing control

This is literally what it says it is - having control over your breathing. You should make sure you are positioned comfortably during this phase of the cycle to enable maximum support and relaxation and, therefore, minimum work by your muscles. You will now be ready to concentrate your mind on breathing control before starting deep breathing. It is important that you read the ‘Breathlessness’ section on page 9 to understand more about breathing control.

Deep breathing

- First, take a long, slow, gentle breath in, through the nose if possible, down to the lower chest (your tummy should rise during this phase, but certainly should not be forced to rise).
- Next, breathe out in a relaxed manner as if sighing out, again not forcing the action. Your chest should be allowed to sag passively. Then continue to try three or four of these breaths before resuming more breathing control.

Huffing

Huffing is a method of clearing sputum from your chest. It uses less energy than coughing and so will make you feel less short of breath. It will also prevent any chest tightness (wheezing) which sometimes occurs after a bout of coughing. If possible, you should try to suppress your cough until you know that sputum is at the back of your throat and ready to be cleared. In order to perform a huff you should:

- keep your chin up to allow your airways to be open, but not so far up that your head is tipped backwards
- take a small half-sized breath in
- open your mouth wide but relaxed in an 'O' shape
- breathe out steadily but quickly through your mouth until you move the sputum.

The huff should be a similar action to steaming up a mirror or your glasses, but remember to have only a small breath in for this technique.

Chest vibrations and clapping (percussion)

These are manual techniques which, when combined with the active cycle of breathing technique (ACBT), will help to free secretions inside your chest, thus enabling you to huff them out more efficiently.

Your physiotherapist will teach you appropriate techniques which you can use yourself or which a partner can perform for you during the deep breathing phase of the ACBT.

However, unless otherwise advised by your doctor or physiotherapist, the techniques should NOT be used if you:

- notice blood in your sputum
- feel very short of breath
- feel wheezy or tight in your chest
- have or are prone to having broken ribs

- better posture
- reduced symptoms of breathlessness and subsequent anxiety
- improved muscle strength which will require less oxygen and therefore put less demand on your lungs
- improved self-confidence and well-being: especially in realising your specific capabilities and limitations.

Okay, so how do I get started?

First of all, do you suffer from any other health problems? If so, you should seek advice from your doctor or physiotherapist before beginning your exercise programme. It is important that the exercise is tailor-made to suit your abilities.

Your programme of exercise should focus on the following:

- the exercises should be progressed gradually - only one step at a time. For example, increase the number of exercise repetitions, the distance you walk or the time spent doing your exercises
- exercise specific areas of weakness. For example, you may have to strengthen leg muscles to help with any difficulty in climbing stairs
- gradually increase your self-confidence by learning to use your breathing control and your own capabilities
- exercise should be tailor-made to a level suitable to you alone. Ask your physiotherapist for advice on how long to carry out each exercise session. A daily, continuing programme designed to bring about steady, gradual improvement is preferable to a once-a-week blitz
- stop exercising if you experience any ill effects such as palpitations, blackout, excessive shortness of breath or pain during or after your activity. It is important to seek advice on symptoms so that they may be investigated. Advice can then be given to enable you to continue an exercise programme.

Some people are afraid to ask about your condition whilst others may expect too much of you. It is often difficult for people to understand when you have nothing to show for your breathlessness, such as a leg in plaster or arm in a sling. People need to be put in the picture.

Remember that it is OK to rest, as long as you have a moderate amount of daily activity. The two go hand in hand. If you find that regular or even one-off rests make your breathing easier, then don't be ashamed to take them. After all, your breathlessness will make you more tired than other people and sleep is vital for your well-being.

- Feelings of tension and anxiety may be eased by different relaxation techniques.
- Some people find that having a partner pat or stoke their back eases their sensation of breathlessness.

Exercise and activity

Since symptoms such as breathlessness and / or sputum production can make you feel anxious, you may not have considered doing any exercise before. Some people are frightened that exercise will make their breathing worse, especially when they already feel short of breath at rest or with minimal activity.

It is important to remember that becoming breathless is a normal response to activity or exercise. Everyone will experience this. However, people who already have certain lung conditions will find that they become breathless more easily (i.e. they don't need to do so much in order to bring on the symptoms). Just make sure you use your practised breathing control to enable you to do the activity or exercise you have chosen.

Regular exercise brings many benefits at times when you are well. A few examples are:

- increased independence in daily activities

- have pain in or around your chest
- have any areas of skin damage around your chest.

Using the ACBT (Active Cycle of Breathing Technique)

The cycle should be practised until your chest is clear and you should change position to drain specific areas of your chest. (Please read the following section on Postural Drainage).

If you become tired or breathless, you may want to rest and continue the breathing cycle later. In addition, the number of daily treatments should correspond to how much sputum you produce. Certainly, during infection, when there is more sputum, you should try to perform chest clearance regularly.

However, even at other times, daily preventive treatments are still important in keeping your airways clear. You may find it easier to build the treatments into your daily routine - perhaps clearing your chest before sleep will ensure a better night's rest.

Chest clearance around meal times is not advisable due to the sensation of nausea after food or loss of appetite before food.

Postural drainage

So what position do I use for the ACBT?

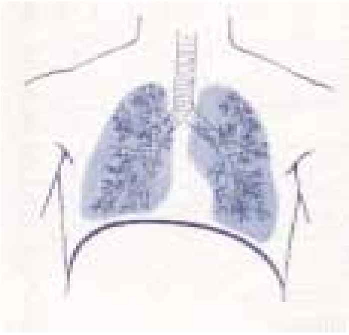

The bronchial tree

Postural drainage means that different postures or positions are used to drain sputum from different parts of the lungs.

The airways in the lungs are often referred to as the 'bronchial tree', since the tubes which they contain originate as one large tube which branches out with offshoots in two directions. These offshoots end as small sacs called 'alveoli'.

A good starting position is sitting relaxed in a supportive chair. You can then progress to lying or sitting in different positions which will allow the variety of lung areas to be drained of sputum using gravity. This technique is called Postural Drainage.

If necessary, your physiotherapist will advise you on which positions to use.

Postural drainage positions

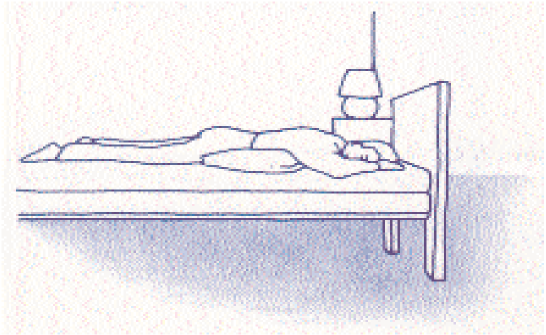

lower lobes

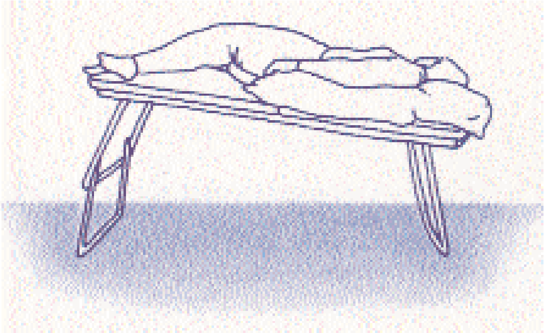

lingula

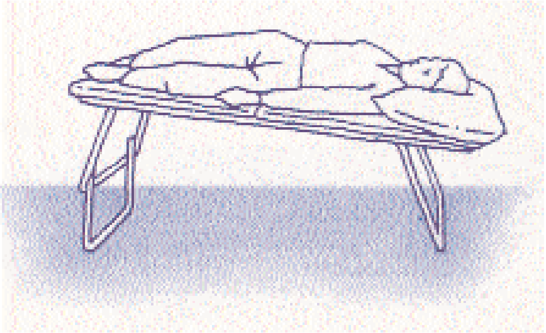

middle lobe

Other tips

- Remember - if you have been prescribed oxygen, it is there to help you and is not harmful.
- Using inhalers when you are breathless can be difficult. Ask your doctor about alternative inhalers which allow you to take time to inhale your medication.
- If your breathlessness limits your ability to carry out normal daily activities such as washing, dressing or cooking, an occupational therapist may be able to help by giving you valuable information and / or equipment for making functional tasks easier to perform.
- Having a door or window open may ease sensations of being warm or claustrophobic and may even reduce your breathlessness.
- Try to be as independent as you can within your capabilities. By keeping as active as possible and increasing your level of activity / exercise you will increase your muscle strength and need less oxygen. This will strengthen your ability to fight further infections (see 'Exercise and activity' on page 13).
- Eating a large meal can seem a daunting task, especially when feeling full afterwards can make you more tired. Try eating little but often or set manageable amounts to eat. Do, however, keep your weight within a normal range. If you are concerned about your nutritional needs, ask to see a dietitian.
- Try to understand your own limitations and set goals which are achievable. This will help you feel more confident in your abilities and help you to be positive in discussing them with people around you.
- Pace yourself in each activity. Don't rush or you will feel exhausted at the end. Use breathing control to help pace your activities.
- Take time to discuss your abilities and disabilities with people close to you. It can be frightening for them too and make them feel helpless.

Breathing control

This is 'controlled' breathing which aims to concentrate your mind on your breathing pattern and enables you to feel 'in control' of your breathing, particularly during attacks of breathlessness.

- First, make sure you are in a comfortable position for practising this technique - try the 'positioning' discussed earlier. This will reduce any unnecessary muscle work, thus reducing the amount of effort you use for breathing.
- Next, breathe at a rate which is comfortable for you - never deliberately try to slow your breathing down - it should, with breathing control, gradually become easier.
- Then, try as much as possible to allow your upper chest to relax and feel supported. Feel the muscles around your neck and shoulders ease.
- Now, focus on your breathing pattern. Remember not to hold your breath but to find a pattern or rhythm of breathing in and out that suits you. For example: try (breathe in), one, (breathe out), two, three, four. Progress as you are able to (breathe in) one, two, (breathe out), three, four, five, six etc.
- Try to keep your upper chest relaxed when breathing in and during this phase allow air to be drawn down to your lower chest and abdomen. Do not force this action, but gently allow your middle to expand.
- You may want to place a hand on the lower part of your chest (just above your tummy button) to feel this or visualise it by sitting in front of a mirror.

You can use this counting method either at rest or even during activity - walking, climbing stairs or during attacks of breathlessness for example - to help you achieve the feeling of being 'in control'. By practising 'breathing control' when you are well, you will be able to use it effectively when you are more breathless.

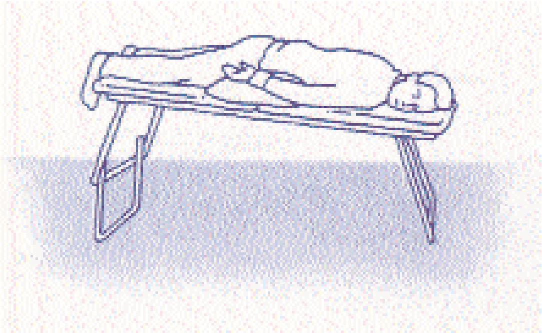

lower lobes

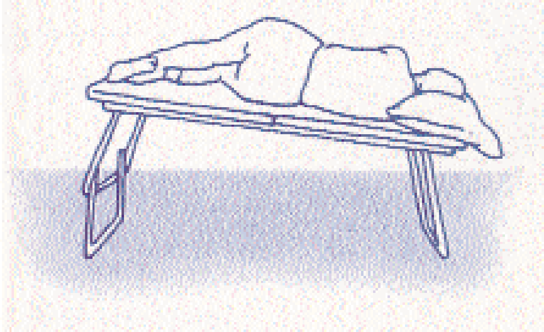

left lower lobe

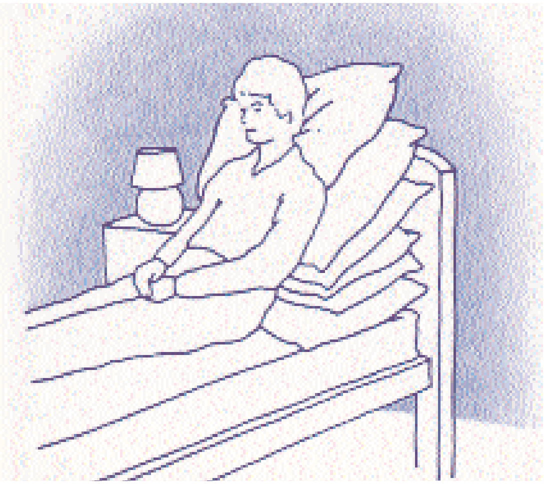

upper lobes

Breathlessness

What is breathlessness?

Breathlessness can be a distressing sensation for people who have specific lung conditions. Becoming breathless with activity or exercise is a normal and natural body response. However, when the lungs are affected by disease, it causes them to function differently and so breathlessness may occur with minor activity or even at rest. Breathlessness may also become more apparent during infections or so-called ‘flare-ups’ or ‘exacerbations’ associated with some conditions.

How will the breathlessness make me feel?

Feeling breathless is completely individual for each person. No one can tell you how you should feel as only you can know!

You may have sensations of being warm, anxious or claustrophobic and may find it easier to breathe by hunching up your shoulders or pursing your lips. All sensations are normal responses, so try not to worry too much about what you ‘should’ or ‘should not’ be doing. Instead, try to focus on small things which you know make your breathing easier to manage.

Why does my lung condition make me breathless?

Many different things can make people feel breathless.

Symptoms such as feeling ‘tight’ or ‘wheezy’, having more sputum in your lungs or being tired are just some examples. Try to find out what causes your breathlessness. Ask your physiotherapist.

What can I do to manage my breathlessness?

Breathlessness is, understandably, a difficult feeling to cope with. For this reason it is valuable for you to feel ‘in control’ of it.

The following suggestions have been designed to help you gain control of your breathlessness and to identify things you can do yourself in addition to things which have been provided medically, such as oxygen or medication.

Positioning

There are certain positions which aid the ‘mechanics’ of breathing in people who are breathless. These positions are adopted during bouts of breathlessness, or during activity, thus easing the symptoms.

The following positions have been found to help the diaphragm muscle work more effectively, enabling your neck and shoulders to be less tense. Positions include:

high side lying

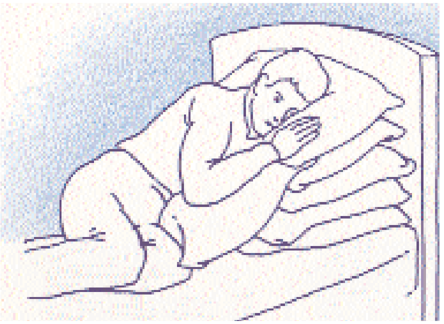

forward lean sitting

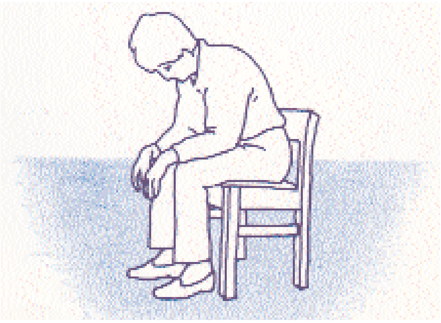

backward lean standing

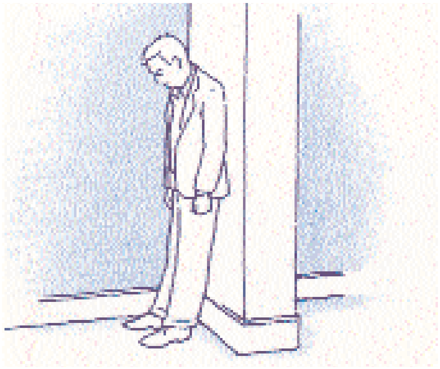

forward lean standing

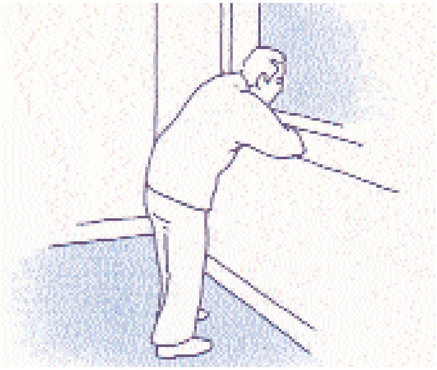

Supplement: Additional file 1 — Help Yourself - physiotherapy for people with respiratory symptoms". Standard booklet given to all patients participating in the trial. [file 1471-2466-10-41-S1.PDF]
