# Supplementary material for: Singing teaching as a therapy for chronic respiratory disease - a randomised controlled trial and qualitative evaluation
Source: BMC Pulm Med. 2010 Aug 3;10:41. doi: 10.1186/1471-2466-10-41 (PMC2920262; doi:10.1186/1471-2466-10-41)
Supplement: Additional file 2 — Singing for breathing - description of program. Detailed description of singing program, selection of materials and conduct of classes. [file 1471-2466-10-41-S2.DOC]

**Appendix Two - Singing for Breathing – description of program**

**1: Lesson overview**

**10 - 20 mins of postural work and physical stretches**

To release muscular tension around areas of the body that link in with the vocal mechanism. As the voice comes from inside the body this covers a wide area, but generally begins with a standing (or seated) position with feet hip width apart, with knees soft and hips not locked, ensuring that muscles in the upper back are not being used to ‘hold up’ the body and gravity is allowing the weight to drop down while the feet root the body and allow an erect skeleton and spine to grow upwards.

Side-stretches are undertaken to release muscles in the intercostals as well as between the shoulder blades and the top of the chest. Hips are rotated to free up the psoas and tranverse abdominus..

Exercises vary depending on individual need but the idea is to ensure that each individual is as free and open as possible to sing with optimum ease.

As an example: one participant from group 5 spent the first eight sessions using all his energy simply to stand up, with his body set into hard tension. By the last session he was transformed – muscular tension was greatly reduced throughout his whole body, including his face. He was able to stand for nearly a whole session and sing through the entirety of a three-minute song, which in the early weeks would be have been impossible.

**10 mins of breath-observation and management**

The workshop leader observes participants breathing normally, in a relaxed fashion. Each participant is encouraged to become aware of how they make their own breathing process harder or easier, i.e. where they are tense, and where they are making space in their body. Some self observation may be involved asking participants to place one hand on their belly and one on their chest to observe the rise and fall of the natural breath. They are then led in some breath exercises which will make singing easier, e.g. allowing the ribcage to release to make more space by engaging muscles in the abdomen (in the waist, under the sternum, and above the pubic bone). These exercises use voiced fricatives such as zzzz or vvvv sounds; once the abdomen is relaxed we gently draw it in (engaging TA’s) to shhhhh and then pitched to shhhhhheeeee. This last encourages prolonged expiration to reduce end expiratory lung volume. The group may also use panting or laughing exercises to involve their abdominal muscles.

**10 mins of vocal exercises**

From staccato arpeggios, for building up abdominal support, to legato arpeggios and melodic phrases (scales), for stamina building using different vowels, plus Brrr and Zzz, which engage abdominal support muscles. The group may also use exercises to improve vocal fold closure (for those with dysphonia) or articulation. This section always ends with ‘extending the count’, i.e. singing to a single pitch on successive numbers from 1 to 16 – as many as possible in one exhalation.

**10 – 20 mins of singing songs**

The extensive list of songs chosen for the Singing for Breathing project covers a wide variety of tempo and pitch so that breath-use can be observed and adjusted.

The primary consideration when working with COPD patients is to extend the out-breath, so songs with long phrases were chosen, such as “Moon River” and “Daisy Daisy” (songs also needed shorter phrases within them to avoid overworking the participant). Songs such as “Drunken Sailor” and “Da Doo Ron Ron” were discarded as they were too fast and did not allow sufficient time for the release and recoil of the in-breath for these compromised patients. Other songs, such as “Kiss Me, Honey Honey”, “King of the Road” or “Sloop John B”, were chosen as they contain pauses more akin to natural speech, helping the participant notice what they do in the pause between breaths. A full list of songs is available on request.

An exercise CD was provided and participants were asked to practice, ideally for 30 minutes each day, either in two 15-minute sessions, or one longer session. It was emphasised that it was more important to practice little and often to a) break unhelpful habits and b) allow the muscle memory to retain the new information. The CD contained a variety of physical warm-ups, relaxation, and vocal and breathing exercises as used in the classes.

**2: Observations**

Until the participant is aware of how their individual postural holding-pattern is impacting upon and potentially limiting their breath use, they cannot make changes. It then takes time for the muscle memory to return to a more relaxed, engaged and focussed breath-use and even longer for the individual to build stamina. My observations of the *general* patterns of learning were as follows:

Lessons 1 – 4 = Development of awareness of individual postural ‘holding’ patterns and for each participant to notice where and how they may be impeding and limiting their optimum breath use through poor postural choices.

Lessons 5 – 8 = Beginning to introduce i) a series of physical exercises to encourage an opening around the muscle groups around the ribcage and those that connect to the diaphragm, ii) breath exercises to allow diaphragmatic and lateral rib-expansion and iii) to extend the exhalation and vocal exercises to different pitches and duration to encourage extended exhalation, and to avoid unnecessary clavicular ‘top-up’ breaths, as well as iv) panting and laughing to free up musculature.

Lessons 9 – 12 = Beginning to build stamina, using more complex pitched exercises and songs with longer phrases at different tempos

The focus overall is on posture, breath and articulation, and less on phonation and resonation.

The Singing for Breathing exercises help patients to:

- Develop awareness of patterns of posture and breath and where they may be unhelpful
- Learn new exercises and let go of old habits
- Develop new ways of producing sound, perhaps through a more ‘natural’ breath-pattern
- Learn new habits through repetition and integration into muscle-memory.

The very act of singing allows breath to deepen, releasing and engaging muscles lower down in the body. This in turn enables longer phrases to be sung and therefore lengthens the exhalation. As patients become aware of what they are doing and begin to make a conscious choice, they will start to manage breathing better.

Over the years the body sets into habitual ‘holding patterns’. As singing comes from inside our bodies, to sing well we need to release these deep-set patterns to express our own ‘sound’ freely. Much of the initial work, therefore, concerns the body and posture. This is not so much about ‘standing up straight’ as getting the body into the best position to allow you to sing with freedom. Years of poor postural habits, exacerbated by breathing issues, may have constricted the muscles around breathing and vocal mechanisms. Particular suspects are muscles around the base of neck, shoulders, upper back and under collarbones and pectoral muscles.

*Singing for Breathing* is more about *not doing* than doing – ideally removing some of the inhibitions which restrict breath-flow ; the aim is to *rediscover* the low breath and accompanying diaphragmatic release we had as babies. For some people with chronic lung disease this may be harder than for others, but even if the diaphragm’s flexibility is reduced, space can be made by moving the intercostals between them and improving lateral or posterial release of the ribcage. Each individual can discover ways of making space within the musculo-skeletal framework to allow the breath to move more freely and then improve stamina through the act of singing.

**3. Practical points for running singing workshops for patients with respiratory disease**

Firstly and fundamentally, singing is about much more than making sound – the lesson will touch on breath-holding patterns which may have become incredibly ingrained over the years, and part of the personality, which may be challenging emotionally and touch on issues around how people perceive themselves, how they work within a group, and how they work with a teacher. Music is emotive and certain songs may give rise to an emotional response. This is about changing habits of a lifetime. Self-esteem will play a part as will cultural and familial attitudes to making noise and emotional expression. Musculo-skeletal change in itself can also be challenging; habits of all kinds are very hard to break.

A balance of activities within a session is important, starting with good relaxation, leading into good body awareness, into physical stretch and opening, to slow engagement of the correct musculature and release of the unnecessary holding, to breath, to spoken word, to slow pitched single notes, to melodic patterns, to songs.

Extensive note taking after each session is imperative for observation and good future planning.

Group cohesion and support encourages better and faster change, as does a good working relationship with the singing teacher

In COPD patients, exacerbations can hit unexpectedly. Factors such as age, fitness, and oxygen-dependency also inevitably affect the singing groups.

Melody, rhythm and the whole singing of patterns in tune and in time may be more challenging for some than for others, and they will be taught in mixed-ability groups.

Singing is not the same as taking a tablet, it is a truly holistic activity which engages the whole person and may prove very challenging for some for all the reasons discussed

Melody, rhythm and the whole singing of patterns in tune and in time may be more challenging for some than for others and they will be taught in mixed ability groups

12 sessions may not be sufficient as longer may be needed to build stamina and engage different muscles. That change will not last without home practice.

Homework is important for success and every group will be different in terms of how it is used used (or not used). This is about changing muscle memory in a way that will not happen effectively without practice. Not everyone practices at home. A practice diary may be useful together with a short booklet and a few vocal exercises. 30 mins a day of practice would likely be appropriate.

It is useful for singing leaders have knowledge of vocal anatomy as well as experience of therapeutic music making and song leading with vulnerable groups.

It is vital that any singing teacher working in a similar setting has good support and clear information around boundaries as well as administrative support, as they will be dealing with a very disparate group of people for whom singing, moving the body and breath work will occasionally raise emotional and psychological issues. Regular feedback meetings may be helpful as well as liaison with clinicians.
