# Supplementary material for: Singing teaching as a therapy for chronic respiratory disease - a randomised controlled trial and qualitative evaluation
Source: BMC Pulm Med. 2010 Aug 3;10:41. doi: 10.1186/1471-2466-10-41 (PMC2920262; doi:10.1186/1471-2466-10-41)
Supplement: Additional file 3 — Open sessions satisfaction survey. Survey used for participants to assess the open singing sessions. [file 1471-2466-10-41-S3.DOC]

# **Your feedback:**

# **Singing for Breathing**

You will have been given this form because you are participating in a new singing project at Royal Brompton Hospital, designed for respiratory patients. As one of the first participants, we would welcome your thoughts, which will help us to understand how to develop our work with singing at the Trust. Please feel free to be as honest as possible! We would like to hear any criticism you may have, as well as anything positive you feel about the idea. Your feedback is confidential and anonymous and your medical care will not be affected in any way.

rb&hArts is a charitable organisation devoted to bringing all forms of the arts to Royal Brompton & Harefield NHS Trust, to improve the well-being of patients, staff and the diverse communities we serve. Singing for Breathing is funded entirely by charitable monies.

**Please return this form to a nurse or send to**

rb&hArts

Royal Brompton & Harefield NHS Trust

Sydney Street

London SW3 6NP

arts@rbht.nhs.uk

1. Have you been to singing lessons/workshops before?

Yes, at school

Yes, as an adult

No

Please circle a number for the following questions:

1. How enjoyable did you find the singing workshop today?

| Not at all enjoyable |  |  |  |  | Very enjoyable |
| --- | --- | --- | --- | --- | --- |
| 0 | 1 | 2 | 3 | 4 | 5 |

3. (a) Do you feel the workshop taught you to think about breathing in a different way?

| Didn’t teach me anything |  |  |  |  | Taught me a lot |
| --- | --- | --- | --- | --- | --- |
| 0 | 1 | 2 | 3 | 4 | 5 |

3. (b) Please give details:

4. (a) Did you feel physically different after the workshop?

| No |  |  |  |  | Yes |
| --- | --- | --- | --- | --- | --- |

4. (b) If yes, please give details:

5. How likely are you to continue singing after today, either with further lessons or at home?

| Not at all likely |  |  |  |  | Very likely |
| --- | --- | --- | --- | --- | --- |
| 0 | 1 | 2 | 3 | 4 | 5 |

6. Did you think that the workshop was: The right length

Too long

Too short

7. Would you like to attend other singing workshops at the hospital? Yes

No

8. Please describe in your own words how you felt about the workshop:

9. Please list any other benefits you felt after taking part today:

10. What changes (if any) to the singing workshops would you suggest?

**Thank you for your time!**
