# Supplementary material for: Singing teaching as a therapy for chronic respiratory disease - a randomised controlled trial and qualitative evaluation
Source: BMC Pulm Med. 2010 Aug 3;10:41. doi: 10.1186/1471-2466-10-41 (PMC2920262; doi:10.1186/1471-2466-10-41)
Supplement: Additional file 4 — Structured interview template. Template used to guide assessments by psychologists following the singing trial. [file 1471-2466-10-41-S4.DOC]

**Appendix 1 - Singing for Breathing - semi structured interview template**

**1) When did you participate in the group?**

**2) When did it end?**

**3) How has the singing group affected you?**

**Prompts: Physically, Emotionally, Behaviourally**

**Benefits? Harms?**

**4) What was the main benefit?**

**5) Were there any unexpected effects good or bad?**

**6) Are you doing anything now that you weren’t before?**

**7) Have you noticed any changes since the end of the group?**

8) **Are you still using singing in your everyday life?**
